# The relative age effect reversal among the National Hockey League elite

**DOI:** 10.1371/journal.pone.0182827

**Published:** 2017-08-14

**Authors:** Luca Fumarco, Benjamin G. Gibbs, Jonathan A. Jarvis, Giambattista Rossi

**Affiliations:** 1 Research Division RED, National Institute of Statistics and Economic Studies (STATEC), Luxembourg, Grand Duchy of Luxembourg; 2 Department of Sociology, Brigham Young University, Provo, Utah, United States of America; 3 Department of Management, Brikbeck, University of London, London, United Kingdom; Bern University of Applied Science, SWITZERLAND

## Abstract

Like many sports in adolescence, junior hockey is organized by age groups. Typically, players born after December 31^st^ are placed in the subsequent age cohort and as a result, will have an age advantage over those players born closer to the end of the year. While this relative age effect (RAE) has been well-established in junior hockey and other professional sports, the long-term impact of this phenomenon is not well understood. Using roster data on North American National Hockey League (NHL) players from the 2008–2009 season to the 2015–2016 season, we document a RAE reversal—players born in the last quarter of the year (October-December) score more and command higher salaries than those born in the first quarter of the year. This reversal is even more pronounced among the NHL “elite.” We find that among players in the 90^th^ percentile of scoring, those born in the last quarter of the year score about 9 more points per season than those born in the first quarter. Likewise, elite players in the 90^th^ percentile of salary who are born in the last quarter of the year earn 51% more pay than players born at the start of the year. Surprisingly, compared to players at the lower end of the performance distribution, the RAE reversal is about three to four times greater among elite players.

## Introduction

Evidence of a Relative Age Effect (RAE) has attracted interest beyond academia due to the fact that seemingly benign policies—such as age cut-offs—may shape later life success [1]. While this phenomenon can be found in multiple studies of hockey [1, 2, 3, 4, 5, 6], most evidence relies on simple metrics to gauge success, such as assessing if there is a disproportionate percentage of hockey players (junior and professional) on a team roster that were born in the first quarter of the year.

Surprisingly, few studies have examined the RAE on player productivity in the National Hockey League (NHL) [7, 8]. In these studies, there is evidence of a RAE reversal. Although players born at the end of the year are less likely to make the NHL, of those who do, they played more games, scored more points, and earned higher salaries [7, 8]. We argue that although the RAE appears to initially favor relatively older players in the minor leagues, if relatively younger players make the NHL, they will likely outperform their peers across a number of outcomes. Thus, being an “underdog” in the minor leagues may lead to improved performance in the NHL.

To date, studies have explored the RAE by averaging the performance of all players. This is reasonable when the data are normally distributed. However, since players born at the end of the year might be disproportiantely placed in the left tail of the ability distribution [2, 3], this approach could underestimate the RAE when elite players are averaged with all other players. To address this possibility, we use a quantile regression to test for the presence of the RAE *along* the distribution of two measures of performance of North American players: total yearly points scored and annual salaries. As noted in previous literature, we expect to find a RAE reversal for both measures. Because of the possible skewed ability distribution, we expect the RAE reversal to be stronger among what we will call the NHL “elite”—players with exceptional talent (as measured by players in the top quantiles of the salary and point distributions) [2].

We also investigate the RAE on the quarter-of-birth distribution on the entire population of North American players. Based on previous literature, we expect players born in the first two quarters to be over-represented, compared to players born later in the year. Moreover, unlike previous studies, we investigate the presence of the RAE on quarter-of-birth distributions by draft age, which is established by NHL drafting rules. In the text, we speculate how these rules may affect the RAE reversal in points and salaries.

## Literature review

### Previous results

There is substantial evidence for the RAE in a number of different contexts, as people born shortly *after* imposed age cut-offs are placed in the subsequent age cohort. As a result, these players can have an age advantage over players born closer to the end of the cut-off. Relative age differences have been found to impact child outcomes such as education [9, 10], self-esteem [11] and physical strength [12]. The RAE can occur for numerous reasons, but in sports, because of the size and maturity advantage for children born right after age cut-offs, relatively older children likely receive more exposure to better competition which contributes to more time for deliberate practice and the development of abilities [5, 6, 13, 14, 15]. In this sense, maturity is mistaken for talent, and those with greater physical maturity are provided more opportunity to train and develop.

Interestingly, when examining the impact of the RAE in sports across a number of sports and countries, results vary. For example, research on German soccer players found that players born shortly after the age cut-off were more likely to play professional soccer [16]. A RAE was also documented for European professional soccer players [17], male and female international basketball players [18], and male and female college volleyball players in Canada [19]. In contrast, the RAE in female sports suggests a more varied pattern than male sports [20, 21, 22, 23, 24]. For example, for females competing in gymnastics and rugby, the RAE appears to be advantageous at younger ages and then have little or no effect in later ages [20, 22]—likely because RAE is sport-specific by gender [24].

Research on the RAE in sports has perhaps been most thoroughly examined among hockey players. Three decades ago, research on players in the NHL and junior hockey (the league that feeds most players to the NHL) found a strong relationship between league participation and birth month [25]. For even younger players (minor hockey), Canadian children born in the first half of the year were more likely to play minor hockey and more likely to play for top teams [6]. More recent research continues to show a strong relationship between birth month and the proportion of players in junior hockey in Canada [3] and the likelihood of being chosen in the NHL draft [2, 5]. Yet, there is growing evidence that the RAE may actually reverse as players advance in professional sports [7, 8]. A reversal has been found in soccer, rugby, handball, cricket, and hockey where relatively younger players appear to suffer disadvantage earlier on, but overcome this disadvantage to earn more money [7, 16], enjoy longer careers [2, 7], score more points [8] and appear on the most elite rosters and squads [2, 26].

### Explanations for the RAE reversal

We highlight two compelling explanations in the literature to understand why a RAE reversal might occur. The first is psychological. Smaller players in junior hockey who subsequently make it to the NHL demonstrate higher than average resilience due to their ability to overcome size limitations [16, 27, 28]. To compete against their relatively older and bigger peers, these players learn to work harder [29], resulting in positive peer effects that spark resilience and improve motivation. This initial disadvantage will eventually work in their favor when early differences in size reach parity in young adulthood. The psychological benefit in the NHL is that these “underdogs” are better equipped to overcome subsequent obstacles and succeed in professional play [28]. Thus, this early disadvantage (if they can overcome it) becomes a later advantage in professional play—an *underdog effect*.

The second explanation suggests that the players born later in the year (relatively younger) who then become successful athletes may not only have a degree of resilience, but also superior ability—a biological explanation. For these younger, smaller players to overcome, “a system that discriminates against them” (372) [16], they need more than grit and determination, they must also be more talented than their relatively larger counterparts to counteract their size disadvantage [16]. It follows that these younger players are likely *positively selected* (i.e. selected from the right tail of the ability distribution). Therefore, while maturity and size can delay or postpone the screening of talented players into the NHL, talent will ultimately win out when assessing performance outcomes. In the NHL, the proportion of relatively young players with superior ability is potentially larger than that of relatively older players because more of the relatively older player’s success has been artificially enhanced by the RAE.

We should note that although these two explanations (effort plus talent) might provide persuasive explanations for a RAE reversal among the NHL elite, there might be a less obvious factor influencing the reversal—the NHL draft age cut-offs. The NHL restricts entry into the draft for players who turn 18 years-old by September 15^th^, but who are not older than 20 years-old before December 31^st^. This means that all 18-year-olds who are drafted in the NHL are born in the first three quarters of the year. Those born in the last quarter of the year must wait another year to enter the draft. Ironically, this means that the same factors that initially benefitted those born in the beginning of the year may reverse their advantage by making them the youngest on their NHL team. For those waiting a year for the NHL eligibility, they will be relatively older than their rookie counterparts. If being slightly older at the start of the NHL career is any advantage, then the initial benefactors of the RAE in junior hockey are now at a disadvantage, simply by another cut-off effect.

Given evidence of the RAE reversal in the literature and compelling explanations for the reversal, we have a simple expectation—the reversal will be greatest among the highest scoring and highest paid athletes in the NHL.

## Data and methods

### Sample

To examine quarter-of-birth distributions and performance outcomes, we compiled data of nearly all NHL players over 8 consecutive NHL seasons; player data across several years were collected from the 2008–2009 season through the 2015–2016 season. This provided a total of 8,760 player-season observations (i.e. 2,017 individual players). Data were collected from two sources, www.nhl.com and www.capfriendly.com. A total of 20 student researchers entered data and a subset of students reviewed the accuracy of the data entered.

We did not use all the player-season observations, but focus on drafted American and Canadian players who were not goalies, and played in a given season. Undrafted players were excluded because draft related information do not exist (e.g. draft year and draft age), which would prevent comparability across models. Likewise, we excluded goalies from the analyses because goalies’ performance outcomes are not comparable to other positions. For a similar reason, observations on players who belong to an NHL team but in a given season played abroad or in minor leagues are excluded because there was no comparable data available when these players are not in the NHL. Finally, the analyses are restricted to North American players because we do not have information on when non-North American players started to play professionally. This totaled 4,447 player-season observations.

We analyzed this selected population with both descriptive statistics and quantile regression analyses [30]. As descriptive statistics can reveal basic patterns in the data, quantile regression can reveal if the RAE varies across the distribution of points and salary, while also accounting for outliers and other statistical issues [31, 32, 33]. We should note that analyses are of all known players in quarter-of-birth-date distribution and does not necessarily require the use of statistical inference, although results with statistical significance scores are still provided in final models (see [34]).

### Analyses of birth dates distributions and NHL drafting rules

To analyze the quarter of birth date distribution, we assess different sub-populations based on age at draft combined with information from the NHL drafting system. To the best of our knowledge, this is the first study to conduct analyses on the RAE on the birth date distribution while accounting for this rule. The NHL’s draft rules establish that only players within a certain age-range in the draft year are eligible for the NHL draft: those who turn 18 years-old by September 15^th^ up until those who turn 20 years-old by December 31^st^ in the draft year. The NHL drafting rules limit relatively younger player’s eligibility period (2 years) compared to relatively older peers (3 years).

### Quantile regression

Quantile regression is an ideal econometric approach to investigate the change of the RAE along the points and salary distributions, yet, only one study has used this technique before within a similar context (on Italian soccer players’ salaries, see [16]). Compared to ordinary least squares (OLS), this method is not limited by the assumption that the RAE is the same at the lower/upper tail of the distribution as at the mean. The quantile regression describes the relationship between measures in the model across quantiles of the outcome variable [33] and is appropriate for studies of outcome variables characterized by extremely positively skewed distributions. This is particularly true for the case when scholars investigate the distribution of athletes’ salaries because of the presence of superstars [31, 32]. Usually, scholars implement a logarithmic transformation of salaries, which we implement as well. However, although the resulting distribution is less skewed, it may still not be sufficiently normal. As a result, OLS might still provide under- or over-estimates.

Quantile regression is also preferred over the utilization of the OLS when this method is used to examine isolated arbitrary sub-samples, based on different outcome levels. When analyzed this way, the investigation of just sub-samples reduces efficiency, since the estimates are obtained from smaller samples. Also, the investigation of sub-samples causes sample selection bias, which occurs with the arbitrary segmentation of the sample into sub-samples [33].

Additionally, the quantile regression could be used as a robustness check to compare the estimates obtained at the conditional median of the outcome variable, that is, the 50^th^ percentile of the distribution, to the estimates obtained at the conditional mean of the outcome variable with the OLS. Thus, while in this study we focus on the quantile regression, we also discuss how these results would change if we used the OLS.

For these quantile regression analyses, we use the Stata 14 command qreg2 [35] and focus on the usual 25^th^, 50^th^, 75^th^, and 90^th^ percentiles of the distribution [15]. This command allows us to compute standard errors clustered on players. Clustered standard errors account for the possibility that the variance of the error term varies by player (i.e. heteroscedasticity), but that it is similar within each player (i.e. in this case a cluster equals an individual player observed over multiple seasons); we use this adjustment because there are repeated observations for individual players that are not likely to be independent. The methodology to compute clustered standard errors for quantile regressions is illustrated in previous research [35].

We conduct robustness checks with four alternative model specifications. First, we conducted analyses on points and salaries with the OLS at the conditional mean. In this model specification, first we insert the quarter of birth dummies and afterwards the other control variables. Second, again with the OLS, we repeat analyses on only Canadian players, and, only for this sub-population, add a dummy variable for players having played in the Canadian Junior Hockey League. In this way, the regression analysis of Canadians works as a an additional robustness check: Canadian and American players trained under the same cut-off (December 31^st^) (except for players who grew up in Minnesota—August 31^st^): the restriction to Canadian players insures the same cut-off date applies to everyone. Third, we repeat the analyses using experience and its standardized square in place of age, as well as season dummies. Fourth, we repeat these analyses on players who were drafted at 19 and 20 years of age.

The Stata syntax for figures and tables in this paper (as well as results reported in the S1 File) are available in the online material. The syntax also includes additional analyses for robustness checks mentioned in this paper, but not reported here.

#### Outcome measures

The outcome measures are annual points (i.e. goals plus assists) and annual salary. In accordance with existing literature, we transform salary data into the natural logarithm. Thus, the RAE estimates on salaries represent salary gaps in percentage terms, with respect to the reference quarter (i.e. January-March). They were computed as:

$$\left[exp\left(\hat{\beta }\right)-1\right]*100$$ (1)

Also, salaries are deflated at 2015 Consumer Price Index For All Urban Consumers (CPI-U) and account for slide contracts, buyouts, and bonuses.

#### Explanatory measures

The key measure of interest is birth quarter. We created dummy variables for quarters of birth, with the aim of capturing possible nonlinear effects [36]. The reference category is the first quarter (January-March). When we analyze birth distribution by quarter, we account for player’s age when the draft occurred. In quantile regressions, we additionally control for the player’s current age, body mass index (weight/(height^2)) (see [37]), player position, season unobservable characteristics, team, country, and draft year. We also account for the standardized square of age ((observed squared age—average squared age)/standard deviation of age; this transformation breaks the collinearity that squared age would have with age).

We should note that over the considered period, mean and median salaries increased monotonically; however, the lack of control for this increase in real salaries does not affect our analyses because this monotonic increase is already captured while controlling for season-specific unobservable characteristics. We should also note that draft age (similar to age at school entry for studies on the RAE in education) is affected by players’ relative age, as suggested in past research [38, 39, 40]. This inclusion as a control variable improves our interpretation of the RAE estimates, as has been adopted in studies on salary discrimination based on ethnicity where performance measures have been analyzed (for a literature review see [41]).

## Results and discussion

### Birth date distribution

**Fig 1:**
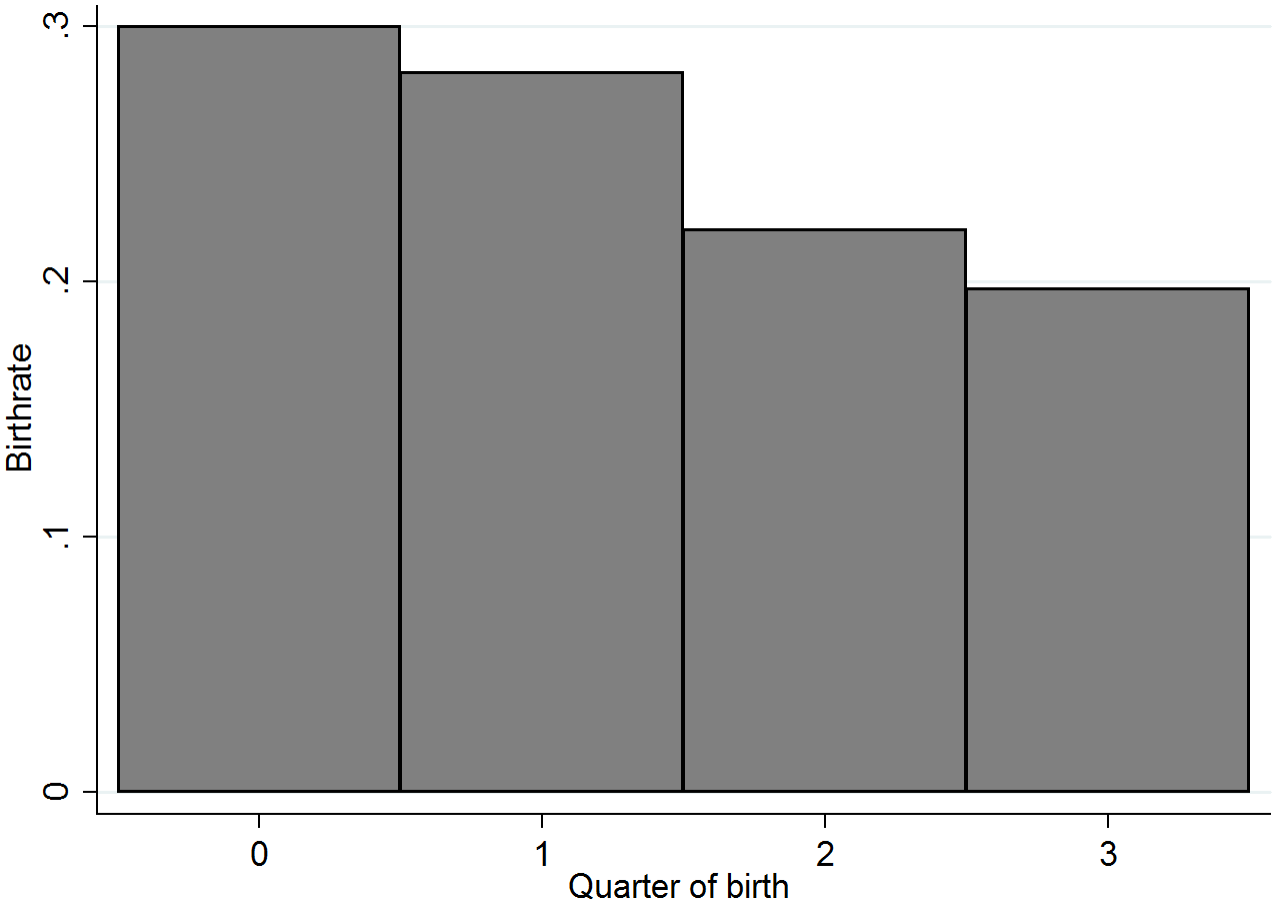
Quarter of birth rate distribution.

**Fig 2:**
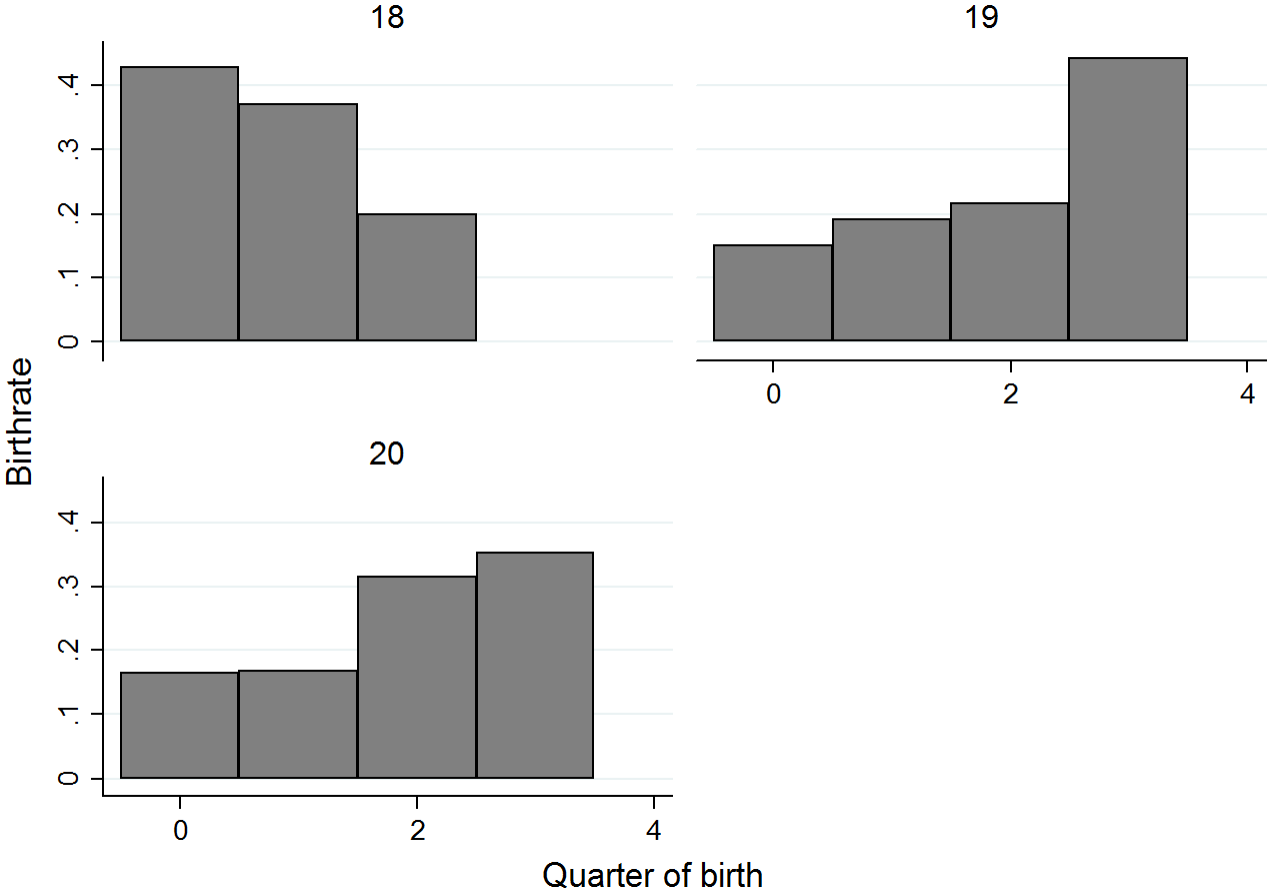
Quarter of birth distributions, based on age at draft.

In this section we analyze the birth date distribution. Fig 1 reports the frequencies of quarter of births for drafted North-American NHL non-goalies (N = 4,447), where January-March is quarter is coded as 0 and so on. October-December is coded as 3. We also investigate the frequency of quarter of birth based on age at draft at 18 (N = 2,363), 19 (N = 1,538) and 20 (N = 546), see Fig 2.

We also illustrate the distributions of quarter of birth for two additional types of players: i) players who play in NHL, but who were not drafted (N = 935)—they entered NHL as free-agents; ii) observations on players who entered NHL (either as draftees or as free-agents), but play abroad or in minor leagues (N = 819). These results are illustrated in S1 File.

Descriptive statistics of the data are presented below. We use pairwise correlation to show the relationship between measures. We also report mean and standard deviation statistics (see Table 1).

**Table 1:** Pairwise correlations and descriptive statistics.

Pairwise correlation^a^						
Variables	**1.**	**2.**	**3.**	**4.**	**5.**	**6.**
**1.** Points	1					
**2.** Ln(salary)	**0.558**	1				
**3.** Age at draft^b^	**-0.071**	**-0.062**	1			
**4.** Age^c^	**0.069**	**0.309**	**0.213**	1		
**5.** BMI	0.012	**0.071**	-0.007	0.149	1	
**6.** Quarter (C)^d^	**0.096**	**0.076**	**0.466**	**0.129**	0.021	1
Descriptive statistics						
N	4,447	4,447	4,447	4,447	4,447	1,447
Mean	19.406	14	0.591	8.332	0.038	1.316
Standard dev.	19.605	1.172	0.698	4.492	0.002	1.101
Min	0	8.059	0	0	0.030	0
Max	109	17.639	2	29	0.053	3

^a^ Correlations in bold are statistically significant at 10%.

^b^ The minimum value of age at draft has been subtracted (e.g., 0 = 18 years of age, 2 = 20 years of age);

^c^ The minimum value of age has been subtracted (e.g., 0 = 18 years of age, 29 = 47 years of age).

^d^(C) stands for “categorical” version of the quarter of birth variable.

Additional descriptive statistics by quarter of birth are provided in Tables 2 and 3. In Table 2, we report the points scored by players in the 25^th^, 50^th^, 75^th^, and 90^th^ percentile of the player-season’s point distribution (upper panel). We do this by quarter and overall. In Table 3 we report equivalent statistics but for salaries.

**Table 2:** Points, by quarter and overall at the 25th, 50th, 75th, 90th percentile.

	Quarter				Overall^a^
	Jan-Mar	Apr-Jun	Jul-Sep	Oct-Dec	
Percentile					
25^th^	3	3	4	4	3
50^th^	11	13	15	15	13
75^th^	25	31	34	34	30
90^th^	54	60	64	60	49
Descr. stat.					
N	1,334	1,254	980	879	4,447
Mean	16.681	19.161	21.773	21.255	19.406
Standard dev.	17.747	19.634	21.266	19.813	19.605
Min	0	0	0	0	0
Max	97	98	109	106	109

^a^“Overall” pulls together observations on players born in different quarters.

**Table 3:** Ln_Salaries, by quarter and overall at the 25th, 50th, 75th, 90th percentile.

	Quarter				Overall^a^
	Jan-Mar	Apr-Jun	Jul-Sep	Oct-Dec	
Percentile					
25^th^	13.411	13.385	13.404	13.459	13.404
50^th^	13.737	13.758	13.81	13.847	13.763
75^th^	14.68	14.914	15.014	15.068	14.923
90^th^	15.548	15.719	15.761	15.703	15.425
Descr. stat.					
N	1,334	1,254	980	879	4,447
Mean	13.873	13.999	14.072	14.112	14
Standard dev.	1.179	1.165	1.181	1.143	1.172
Min	8.09	8.059	8.102	8.102	8.059
Max	16.474	17.639	16.486	16.811	17.639

^a^“Overall” pulls together observations on players born in different quarters.

**Fig 3:**
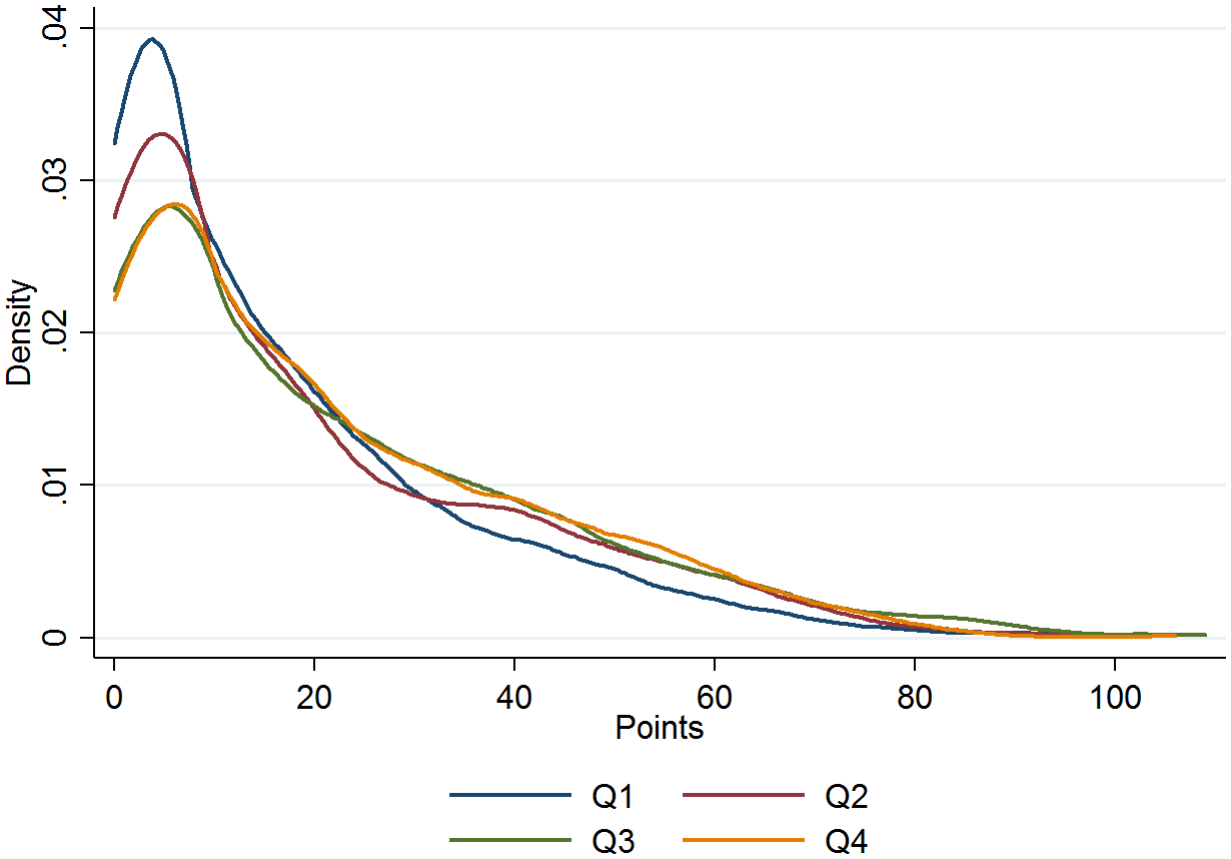
Points distribution, by quarter of birth.

**Fig 4:**
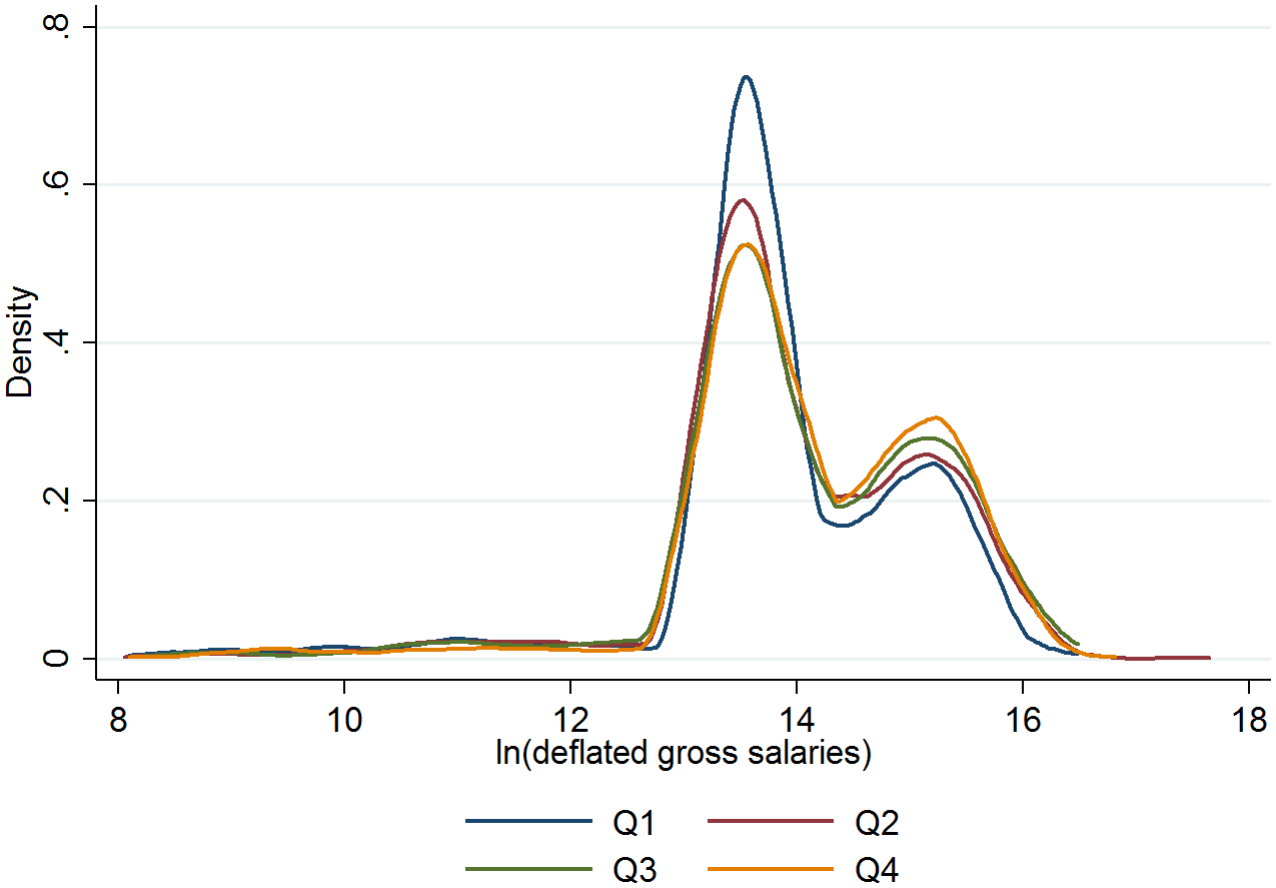
Salaries distribution, by quarter of birth.

Figs 3 and 4 provide a visual distribution of scores and salary by birth quarter.

### Points

In this section we investigate the RAE on points using quantile regression (as a point of reference, see S1 File for OLS results). We focus on the 25^th^, 50^th^, 75^th^ and 90^th^ percentiles of the points distribution of North American players. The results are reported in Table 4. Analyses with the conditional mean and only for Canadian players using OLS is reported in the supporting material (see Table A in S1 File).

**Table 4:** The RAE by quarter, on points; quantile regression at the 25th, 50th, 75th, 90th percentile.

Variables	North Am.^a^ Points 25%	North Am. Points 50%	North Am. Points 75%	North Am. Points 90%
April-June	0.116	0.452	3.135	0.956
	(0.540)^b^	(1.443)	(2.723)	(2.737)
July-September	1.122	4.869**	7.981***	6.333**
	(0.727)	(2.035)	(2.647)	(2.786)
October-December	1.819**^d^	6.546***	11.46***	9.222***
	(0.854)	(1.987)	(3.072)	(2.885)
Control variables	Y	Y	Y	Y
Can. Jr. Hockey	N	N	N	N
Observations	4,447^c^	4,447	4,447	4,447
Pseudo R-squared	0.058	0.120	0.132	0.114

^a^ Only North American players are investigated.

^b^ Standard errors in parenthesis are clustered on players.

^c^ Repeated observations per player are used.

^d^ *** p<0.01,** p<0.05,* p<0.1.

We conducted additional analyses not reported for brevity, but available upon request. First, we control for experience, its standardized square in place of age, and season dummies; the results are equivalent to those in Table 4. Second, we restricted the analyses on players who were drafted at 19 and 20 years of age; these results point in the same direction of those in Table 4, and are even stronger because the best players who were drafted at 18 years of age are excluded.

### Salaries

In this section we investigate the RAE on salaries. As with points, we implement the analyses at the 25^th^, 50^th^, 75^th^ and 90^th^ percentiles of the salary distribution of North American players.

As before, OLS analyses of ln(salary), including that with the restricted sample on Canadian players alone, can be found in the supporting material (see Table B in S1 File). Results controlling for experience, its standardized square in place of age, and season dummies, as well as analyses on players drafted at 19 and 20 years of age are not reported for sake of brevity (available upon request). The results from these additional analyses confirm those in Table 5.

**Table 5:** The RAE by quarter, on natural logarithm of salaries; quantile regression.

Variables	North Am.^a^ Ln_Salary 25%	North Am. Ln_Salary 50%	North Am. Ln_Salary 75%	North Am. Ln_Salary 90%
April-June	-0.019	0.042	0.041	0.064
	(0.043)^b^	(0.060)	(0.091)	(0.083)
July-September	0.064	0.194**	0.217**	0.166*
	(0.050)	(0.077)	(0.093)	(0.086)
October-December	0.149**^d^	0.289***	0.392***	0.414***
	(0.066)	(0.083)	(0.112)	(0.110)
Control variables	Y	Y	Y	Y
Can. Jr. Hockey	N	N	N	N
Observations	4,447^c^	4,447	4,447	4,447
Pseudo R-squared	0.125	0.172	0.158	0.128

^a^ Only North American players are investigated.

^b^ Standard errors in parenthesis are clustered on players.

^c^ Repeated observations per player are used.

^d^ *** p<0.01,** p<0.05,* p<0.1.

## Discussion

With the available literature as our guide, we had hypothesized that the reversal will be greatest among the highest scoring and highest paid athletes in the NHL. We find that Fig 1 provides prima facie evidence that players born in the third and fourth quarter are under-represented, since they should represent approximately 25% of the distribution each. Fig 2 shows the quarter-of-birth distribution by age at draft. As per the rule, this figure shows that no player born in the fourth quarter is drafted at 18; in contrast, at 19 and 20 years of age about 45% and 38% of the players respectively are born in the fourth quarter. While NHL teams prefer to draft 18-year old players, players born in the fourth quarter of their 18^th^ year cannot be drafted. Therefore, although we observe a RAE reversal on the distributions of quarter of birth for players drafted at 19 and 20, there is no reversal possible in the overall distribution of quarters of birth. On one hand, this result is important because it shows a mechanic inflation of the under-representation of relatively young players. On the other hand, this result might provide an additional explanation for the RAE reversal in terms of performance; more details are discussed in the next section.

We should note that, as Fig A in S1 File suggests, being selected as a free-agent (and thus enter later into NHL) does not contribute to the overall under-representation of relatively young players. In fact, this figure shows an approximately uniform distribution of quarters of birth for free-agents.

Finally, in line with expectations, we observe an over-representation of players born in the first two quarters among non-NHL players (e.g. players that belong to NHL teams but in that season are loaned to minor leagues or teams abroad) (see Fig B in S1 File). This figure suggests that relatively older players who could enter the NHL when they were 18 years-old are more frequently considered too immature to play in the NHL and are sent to minor leagues to cumulate experience. This analysis was also only conducted on non-NHL players who were drafted—a slightly smaller sub-sample; this result is not reported for sake of brevity (available upon request).

Table 1 shows that quarter of birth is positively and statistically significantly correlated to both points and salaries; this is confirmed in Tables 2 and 3. For points, the first-quarter player in the 90^th^ percentile scored 54 points in a given season, while the player born at the end of the year scored about 60 points (see Table 2). Likewise, Table 3 on the natural log of salaries for selected players along the distribution suggests a RAE reversal. Figs 3 and 4, on the distribution of salaries and points respectively, by quarter of birth, provide a graphic illustration of the RAE reversal.

Table 4 provides evidence of the RAE reversal that is greatest among NHL elites in terms of points. We see that in no percentile do the players born later in the year score less than their older peers born earlier in the year. The RAE reversal increases in the quantile of the points distribution: the positive points gap in favor of athletes born later in the year is small, being 1.8; then it increases at the median by almost four times, and at 75% and 90% of the points distribution, relatively young players score 11.4 and 9.2 more points than their relatively older counterparts. Also, athletes born in the third quarter enjoy a positive points gap, which ranges between about 5 and 8 additional points and appears only from the median of the points distribution.

Also, we find strong evidence of the RAE in terms of salaries (see Table 5). In none of the percentiles do the players born later in the year earn less than their older peers born earlier in the year. Moreover, we observe that the RAE reversal is driven by salary disparities in the top quantiles. The positive salary gap in favor of athletes born later in the year is small (16%) then increases at the median salary, where players born in the fourth quarter earn 33.5% more than players born in the first quarter. At 75% and 90^%^ of the salary distribution, relatively young players earn about 48–51.3% more than relatively older counterparts. Also athletes born in the third quarter enjoy a positive salary gap, but it is smaller and does not seem to increase by quantile in a consistent way.

Results replicated with the OLS (see Tables A and B in S1 File) at the conditional mean of the outcome variable tend to be quite larger than those at the median of the outcome variable with the quantile regression. Therefore, as expected, results are less sensitive to variations in point and salary gaps across the distribution. Results from additional robustness checks provide results in the same directions.

### Three elite players

To illustrate our findings, we compare our results with three NHL “elite” players born in different quarters of the year. The following players, Steven Stamkos, Sydney Crosby, and Patrick Kane, are the highest scoring North American players in the NHL and have the highest points per game average for all North American players over the period we cover in our data (since the 2008–2009 season). Steven Stamkos was born in the first birth quarter (February) and has averaged 65 points per season over his career, which is 15 points higher than the points scored by players in the 90^th^ percentile of the overall distribution. Sydney Crosby, born in the third birth quarter (August), has averaged 85.5 points per year, which is about 46 points higher than the 90^th^ percentile. Finally, Patrick Kane, born in the fourth birth quarter (November), has averaged 75 points per year throughout his career, which is about 36 points higher than the 90^th^ percentile.

Their career average salaries reflect their exceptional productivity *above* the 90^th^ percentile of the overall distribution. Steven Stamkos, again born in the first quarter, has an average salary of $5,912,500 per season (a 15.59 on the natural logarithmic scale). Sidney Crosby, born in the third birth quarter, has an average salary of $9,286,364 (a 16.04 on the natural logarithmic scale). Patrick Kane, born in the last birth quarter, has an annual salary of $6,865,909 per year (a 15.74 on the natural logarithmic scale). We see that they are all exceptionally compensated for their productivity at levels much higher than even the top 10 percent of the overall distribution, even as the patterns still reflect a RAE reversal. Although not always true for each individual case, from these comparisons we are able to illustrate what our findings show—a RAE reversal that is more pronounced among the NHL elite.

### Limitations

This investigation presents at least two limitations. First, our study is based on data from only two countries: US and Canada. This limits the external validity of our results. Second, these countries share the same cut-off date (except for the state of Minnesota), which could give rise to one problem—we cannot disentangle the RAE from season-of-birth effects, which are shown to exist in the educational system in the US and that could also affect youth hockey teams in the US and elsewhere. These season-of-birth effects are potential confounders because they might impact the performance of players born in different periods of the same calendar year in unknown ways. This effect would be independent from maturity gaps in youth as one study suggests [42]—US winter-children are disproportionately born to single mothers, teenage mothers and mothers without a high-school degree. The impact of family structure could cause negative season-of-birth effects on children, at least for school achievement. Likewise, this could also impact the composition of future hockey players born in winter months. Thus, this type of season-of-birth effect could bias results.

## Conclusions and future directions

We advance the RAE literature in two ways. First, we test for the presence of the RAE on points and on salaries with quantile regressions, which allow us to explore how the RAE varies along the distribution of points scored and salary. Second, we investigate the RAE on the quarter of birth distribution by draft age (i.e., 17, 18, 19), which is established by NHL drafting rules; this is the first time such analysis is conducted.

Overall, we find evidence of a RAE reversal in terms of both points and salaries. In each season, players born in the fourth quarter scored more points and earned more than players born at the beginning of the year. These gaps increased approaching the top quantiles of the points and salaries distributions, and these results could be explained by the previous literature that suggests the possible role of resilience and selection on talent. As initially suspected, conventional methods to analyse the RAE lead to over-estimates of the RAE, compared to those from quantile regression at the median value of the outcome variable.

Additionally, as expected, we find that players born in the third and fourth quarter are under-represented. However, unlike previous studies, we show that this distribution changes by age at draft: no player born in the fourth quarter is drafted at 18; in contrast, at 19 and 20 years of age at draft, about 40% of the players are born in the fourth quarter. Moreover, we observe that relatively older players drafted at 18 years-old are more frequently sent to the minor leagues. Given the combination of NHL drafting rules and the preference of NHL teams to draft 18-years-old players, the under-representation of relatively young players is mechanically inflated. However, this delayed entry of relatively young players into the NHL may eventually benefit them—in terms of performance. When they are drafted they may have accumulated more playing-time than older peers who have already been drafted at 18 by an NHL team, but might have played a lower amount of time in their first NHL season. This additional on-ice time before reaching the NHL may provide an edge in terms of performance (and thus wages) to fourth-quarter players during their professional career. This interpretation on the positive effect of a delayed draft on performances is compatible with evidence from the National Football League [36].

In total, our findings suggest that future work should explore potential psychological and biological factors that may account for the RAE reversal, *especially among the NHL elite*. In this way, our results also suggest an exploration of possible mediating factors that explain why elites have such pronounced reversals in the RAE. To offset the inherent unfairness of the RAE and the later RAE reversal on performances, understanding these mediating factors could be used to better select talent and encourage resiliency of players. Although previous literature on the RAE in sports and education [38, 39, 40] suggests delaying entry into professional sports/school to reduce performance gaps, the reversal complicates this view as there are potential benefits of being the “underdog.” Finally, from the perspective of the RAE in term of representativeness, our results suggest that adjustments of the drafting rules could reduce the disadvantages suffered by relatively young players.

Future studies could analyse performance of players from countries with different cut-off dates, but who play in the same tournament (e.g. the world championship or the Olympic winter games). In this case, researchers would need to collect harder-to-find data on international players, such as age at entry into professional hockey and other factors. Second, future studies could examine variations of the age cut-off date within a single country, as it is done in soccer [43, 44]. Overall, we think the future of RAE research is clear—the RAE and its reversal reveal how critical something as arbitrary as age cutoffs can be for sport performance and suggests a complex interplay of ability, phycological and social contexts in understanding success in the NHL and beyond.

## Supporting information

S1 File Fig A) Quarter of birth distributions of free-agents. Fig B) Quarter of birth distributions of non-NHL players. Table A) RAE by quarter, on points; OLS. Table B) RAE by quarter, on natural logarithm of salaries; OLS.
(DOCX)

S2 File Analyses. This is the Stata 14 code used to analyze the data.
(DO) Click here for additional data file.

S3 File Dataset. This is the complete Stata dataset used for the investigations conducted in this paper.
(DTA)
