# Supplementary material for: The relative age effect reversal among the National Hockey League elite
Source: PLoS One. 2017 Aug 14;12(8):e0182827. doi: 10.1371/journal.pone.0182827 (PMC5555707; doi:10.1371/journal.pone.0182827)
Supplement: S1 File — Fig A) Quarter of birth distributions of free-agents. Fig B) Quarter of birth distributions of non-NHL players. Table A) RAE by quarter, on points; OLS. Table B) RAE by quarter, on natural logarithm of salaries; OLS. (DOCX) [file pone.0182827.s001.docx]

**S1 File**

**Fig A Quarter of birth distributions of free-agents.**


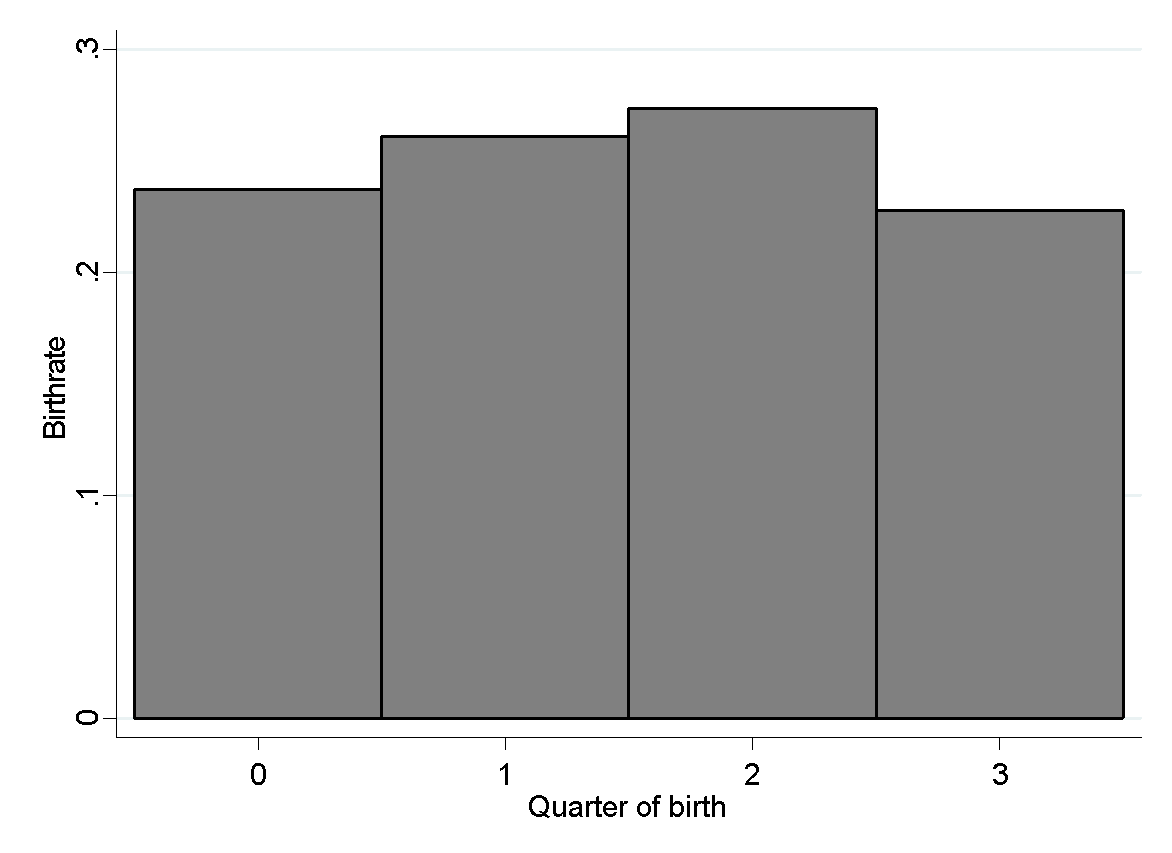


**Fig B Quarter of birth distributions of non-NHL players.**


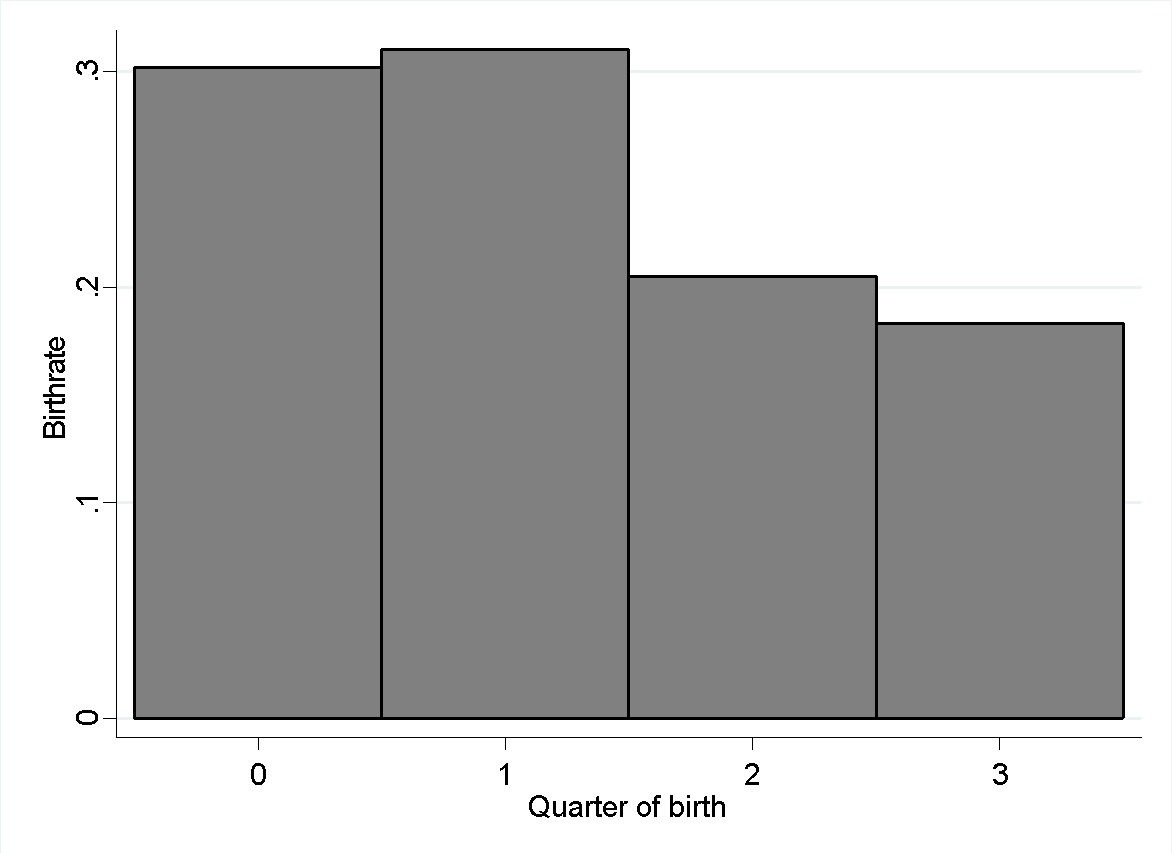


Table A. RAE by quarter, on points; OLS.

|  | North.-Am.^a^ | North.-Am. | Canadians^d^ |
| --- | --- | --- | --- |
| Variables | Points | Points | Points |
|  | (1) | (2) | (3) |
|  |  |  |  |
| April-June | 2.480 | 2.176 | 3.215* |
|  | (1.549)^c^ | (1.438) | (1.704) |
| July-September | 5.093*** | 6.221*** | 6.905*** |
|  | (1.786) | (1.666) | (1.981) |
| October-December | 4.574*** | 8.538*** | 7.414*** |
|  | (1.745) | (1.795) | (2.090) |
|  |  |  |  |
| Control variables | N | Y | Y |
| Can. Jr. Hockey | N | N | Y |
|  |  |  |  |
| Observations | 4,447^d^ | 4,447 | 3,103 |
| R-squared | 0.011 | 0.142 | 0.175 |

$$*** p<0.01, ** p<0.05, * p<0.1.$$

^a^ Only North American players are investigated.

^b^ Only Canadian players are investigated.

^c^ Standard errors in parenthesis are clustered on players.

^d^ Repeated observations per player are used.

Table B. RAE by quarter, on natural logarithm of salaries; OLS.

|  | North.-Am.^a^ | North.-Am. | Canadians^b^ |
| --- | --- | --- | --- |
| Variables | Ln_Salary | Ln_Salary | Ln_Salary |
|  | (1) | (2) | (3) |
|  |  |  |  |
| April-June | 0.126 | 0.087 | 0.134 |
|  | (0.087)^c^ | (0.076) | (0.088) |
| July-September | 0.199** | 0.260*** | 0.326*** |
|  | (0.094) | (0.087) | (0.102) |
| October-December | 0.239** | 0.412*** | 0.390*** |
|  | (0.093) | (0.099) | (0.122) |
|  |  |  |  |
| Control variables | N | Y | Y |
| Can. Jr. Hockey | N | N | Y |
|  |  |  |  |
| Observations | 4,447^d^ | 4,447 | 3,103 |
| R-squared | 0.011 | 0.125 | 0.159 |

$$*** p<0.01, ** p<0.05, * p<0.1.$$

^a^ Only North American players are investigated.

^b^ Only Canadian players are investigated.

^c^ Standard errors in parenthesis are clustered on players.

^d^ Repeated observations per player are used.
